# A common cause for a common phenotype: The gatekeeper hypothesis in fetal programming

**DOI:** 10.1016/j.mehy.2011.09.047

**Published:** 2012-01

**Authors:** S. McMullen, S.C. Langley-Evans, L. Gambling, C. Lang, A. Swali, H.J. McArdle

**Affiliations:** a The Rowett Institute of Nutrition and Health, University of Aberdeen, Greenburn Road, Bucksburn, Aberdeen AB21 9SB, UK; b School of Biosciences, University of Nottingham, Sutton Bonington, Loughborough LE12 5RD, UK

## Abstract

Sub-optimal nutrition during pregnancy has been shown to have long-term effects on the health of offspring in both humans and animals. The most common outcomes of such programming are hypertension, obesity, dyslipidaemia and insulin resistance. This spectrum of disorders, collectively known as metabolic syndrome, appears to be the consequence of nutritional insult during early development, irrespective of the nutritional stress experienced. For example, diets low in protein diet, high in fat, or deficient in iron are all associated with programming of cardiovascular and metabolic disorders when fed during rat pregnancy. In this paper, we hypothesise that the nutritional stresses act on genes or gene pathways common to all of the insults. We have termed these genes and/or gene pathways the “gatekeepers” and hence developed the “gatekeeper hypothesis”. In this paper, we examine the background to the hypothesis and postulate some possible mechanisms or pathways that may constitute programming gatekeepers.

## The theory of programming

During pregnancy, the developing fetus is entirely dependent on its mother for nutritional requirements. It is axiomatic, therefore, that maternal nutrition will have a very important role to play in growth, development and pregnancy outcome. This role extends beyond the fetal and neonatal periods and into adult life. Many studies, in both humans and animals, have demonstrated that sub-optimal nutrition during pregnancy can have marked consequences for the offspring, even as an adult. These observations led to a hypothesis known as the “fetal programming” hypothesis [1]. Programming may be defined as the response made by an organism to an insult or stimulus occurring during a critical period of development. As developmental processes occur in a defined sequence, adaptations that occur in response to external signals during phases of growth or maturation will generally be irreversible. Hence the environment can have a permanent impact on physiology and long-term health and well-being.

### Evidence from epidemiological studies

The basis for the fetal programming hypothesis was a series of retrospective cohort studies considering the health of men and women born in the early part of the twentieth century, in the UK, Finland and Sweden. Originally, the data suggested that the link was primarily with weight at birth, as numerous studies described the association between low birth weight and cardiovascular morbidity and mortality [2–5]. Critically, this association was seen within the normal range of birth weight, not just in infants that are born small [6]. Barker’s Hertfordshire cohort, a UK population born between 1910 and 1930, for example showed graded and linear relationships between birth weight and coronary heart disease death, blood pressure, type 2 diabetes and the metabolic syndrome [2,4,7,8].

More recently it has been suggested that other parameters may also be relevant in determining risk of disease in the offspring. The most robust of these may be the ratio between the placenta and the newborn infant, which gives an indication of placental efficiency [9]. A large US cohort study demonstrated that a high placental-to-birth weight ratio, but not birth weight itself, was associated with high blood pressure in childhood [10]. Similarly, a Norwegian cohort study showed a positive association between placental-to-birth weight ratio and cardiovascular disease [11]. Evidence also suggests that growth in the first year of extra-uterine life is important. A number of systematic reviews have supported the concept that increased growth rate in early life is a risk factor for subsequent obesity. Upwards crossing of centiles for weight and length in infancy is associated with later obesity risk [12,13] and it has been estimated that 20% of the risk of obesity at 7 years of age can be attributed to being in the highest quintile for weight gain over the first 4 months of life [14]. Studies of adults born in Helsinki in the first half of the twentieth century suggest that individuals who go onto suffer from coronary heart disease were smaller at birth but gained weight rapidly in childhood [3]. While early life experience appears to be an important factor determining risk of non-communicable disease in adult life, the overall impact of programming will be dependent upon other risk factors. For example, other work indicates that there are interactions between early life factors and adult lifestyle, with the greatest risk of metabolic disorders associated with relative thinness at birth and obesity in adulthood [15]. In addition to these interactions between early environment and adult risk factors, programming influences appear to interact with genotype. For example, it is reported that there is an interaction of the pro12ala polymorphism of PPAR γ-2, with markers of prenatal growth, in determining adult insulin sensitivity, cholesterol metabolism and risk of cardiovascular disease. The Ala12 allele is associated with significantly lower fasting insulin and HOMA-IR index, but this beneficial effect of the polymorphism is isolated to individuals who were of lower weight at birth [16].

### Evidence from animal models

While such epidemiological studies have played a key role in highlighting the associations between early life events and later disease, they are limited in their ability to identify the causal factors that underpin early life programming and have been frequently criticised on the basis of inadequate adjustment for confounding factors and inconsistent study designs, outcomes and interpretation [17]. Experimental studies using animals have played a critical role in demonstrating the biological plausibility of the fetal programming hypothesis and are now being used to examine the mechanistic drivers of such programming. Evidence of prenatal programming of adult physiological function and disease by maternal nutritional status has been demonstrated and replicated using a broad range of animal models.

Animal studies that have investigated the early life programming of adult cardiovascular disease have used maternal dietary manipulations to influence fetal growth. It is consistently noted in rats [18,19], mice [20] and guinea pigs [21] that fetal exposure to undernutrition produces elevated blood pressure. Similar observations in large animal species such as the sheep [22,23], suggest that programming of cardiovascular function occurs in all mammals. The nature of the nutritional insult does not appear to be specific as it is noted that imposing a wide variety of nutritional and environmental stressors during pregnancy produces the same general phenotypic outcomes in the offspring. Hypertension is noted in the offspring of rodents exposed to maternal food restriction [19], protein restriction [24], iron deficiency [25], high fat feeding [26], uterine artery ligation [27] and dexamethasone treatment [28]. The same range of maternal treatments is also associated with impaired glucose homeostasis, renal impairments and metabolic disturbance in the offspring.

## The thrifty phenotype hypothesis

Following the development of the fetal programming hypothesis, the “thrifty phenotype” hypothesis was proposed by Hales and Barker [29]. This argued that development *in utero* is regulated in a way which sets the metabolism of the developing fetus to be optimal within a certain postnatal nutritional environment. The thrifty phenotype hypothesis proposed that fetal exposure to undernutrition resulted in metabolic adaptation that encouraged efficient (thrifty) utilization of scarce energy substrates. If the same nutrient- or energy-poor environment prevailed in the postnatal period, then the individual may be conferred with metabolic advantage. Difficulties would arise if the postnatal nutritional environment did not match the fetal environment (i.e. relative excess of energy and nutrients in the postnatal period), as the individual would be irreversibly maladapted for such conditions. This hypothesis was initially proposed to explain the development of insulin resistance [29] but has since been extrapolated to other aspects of metabolic syndrome. Whether this extension is justified is not completely clear.

One such population where this hypothesis was tested was the group of Ethiopian Jews (Falasha) who migrated from the Gondar region of Ethiopia to Israel in the 1980s. Cohen and colleagues [30] first reported that among young Ethiopian-born men who had been living in Israel for less than 4 years, there had been a major shift in dietary habits. Rather that consuming a diet based upon the Ethiopian injura bread and spicy stews, the Falasha migrants took on a westernized diet rich in refined carbohydrate sources. Remarkably the prevalence rates for diabetes in this population soared to 18%, some 30-fold higher than among the original Ethiopian population and 2-fold higher than among other ethnic groups in Israel. It could be argued that this shift demonstrates the thrifty phenotype in action. The Falasha migrants while *in utero* would have developed in an environment of scarce resource, and whilst remaining in their environment their acquired metabolic thrift would have been advantageous. The shift to the Israeli pattern of diet, however, brought out the negative consequences of that thrift. An alternative explanation is that the Falasha carry a genotype that promotes diabetes, but only when individuals are exposed to a diabetogenic environment [31].

In animal models, alterations in glucose homeostasis and insulin sensitivity which occur in response to prenatal undernutrition appear to fit with this theory. For example, offspring of the uterine ligation model exhibit reduced beta cell mass [32] and relative hyperglycaemia [33]. Maternal calorie restriction also leads to hyperinsulinaemia in adulthood in the rat [34].

The thrifty phenotype hypothesis has been developed further to suggest that it is only one of a series of adaptations that occur during development to a predicted external environment. The “predictive adaptive response” theory argues that there are selective advantages in predicting the environment into which an animal will be born and preparing appropriately [35]. Using examples from developmental zoology, the authors of this paper propose that plasticity in response to nutrition is only one of a series of modifications that can be, and are, made. For example, voles will have different degrees of fur covering at birth depending on the time of year they are born [36].

The thrifty phenotype hypothesis and the predictive adaptive response theory appear to fit with many observations from epidemiology and animal experiments. However, there are clear examples of studies where they cannot explain observed consequences of variation in maternal nutrition. The main thrust of these hypotheses is that disease is the consequence of a mismatch between fetal and postnatal environments. However, it is clear that in the case of animals exposed to maternal high fat feeding or obesity, the “match” of environments whereby the animals are also weaned onto a high fat, obesogenic diet, produces the most detrimental effect on health [37–39].

## The response to intrauterine insult: mechanisms of programming

All of the hypotheses outlined above have considerable value. They are all testable and also imply ways of alleviating the problems caused by suboptimal intrauterine nutrition. However, they do not suggest possible mechanisms whereby the phenotype is generated. They also do not explain why, even though the stresses imposed are of many different types, the phenotype that is generated is remarkably similar.

The majority of programming manifests itself as many of the constituent parts of the metabolic syndrome, characterised by hypertension, obesity, diabetes and dyslipidaemia [40,41]. Irrespective of the stress, whether it is low protein, low iron, high fat or some other effector, the offspring manifest at least three of the four symptoms. Within the current theoretical frameworks it is not clear why apparently diverse nutritional insults result in the same common pattern of programmed responses. There are two strands to consider in unravelling the mechanisms of programming. Firstly it is important to understand the changes which occur in the embryo, fetus or neonate at the time of the actual insult as these will be the primary drivers of the programmed phenotype. Secondary to this there will be mechanistic changes which lie on the main route from this initial response to compromised physiological function and disease pathology. It is in this latter area that the greatest concentration of experimental evidence from animal studies has been focused.

The simplest explanation of nutritional programming relates to altered organ and tissue structure, modifying the capability of organs to perform normally. Organs develop from progenitor stem cells, which have a strict order of growth and development. They also grow at tightly specified times in development. Anything that disrupts this order is very likely to have consequences [42], and in the simplest scenarios will result in organs that are smaller, due to a lower final cell number, or which are morphologically altered due to the presence of alternative cell types. This process has been termed tissue remodelling [43]. The tissue remodelling phenomenon has mostly been studied in kidney, by our group and others [44,45]. Feeding a low protein diet during rat pregnancy, particularly during the later stages of gestation, results in a kidney of normal size but with a reduced (up to 30%) complement of nephrons. Similar observations have been made in sheep following maternal food restriction during the major phase of nephrogenesis [23]. Most importantly, this remodelling aspect of programming has also been suggested in human patients exhibiting symptoms of primary hypertension [46]. Although as yet not extensively investigated, the brain [47], pancreas [48], hypothalamus [49], muscle [50] and placenta [51–54] also show evidence of remodelling which may affect organ function.

Relating the remodelled tissue morphology to tissue and organ function and the capacity of the organ to maintain physiological competence throughout the lifespan is a key component of being able to explain how programming leads to disease. A reduction in nephron number, for example, is proposed to be one of the drivers of systemic hypertension. The nephron is the basic functional unit of the kidney and with a lower nephron complement local blood pressure has to be increased to maintain filtration function. Over time this leads to further nephron loss, glomerular injury [55] and rising local blood pressure eventually manifests as systemic hypertension. Similarly remodelling of neuronal densities in the appetite regulation centres of the hypothalamus [49] may explain how fetal exposure to maternal protein restriction impacts upon feeding behaviours.

Evidence of programming of the hypothalamic control of feeding provides an interesting perspective on other routes leading from early insult to later disease. Remodelling of tissues with critical homeostatic functions could effectively “reset” aspects of physiology, particularly if the affected tissues are endocrine organs or if programming has impacted upon the cells which are the targets for endocrine signalling. The renin–angiotensin system is a multi-organ system that regulates blood pressure and hypertension is one of the most common outcomes of nutritional programming. The kidney secretes the enzyme renin, which cleaves the liver produced angiotensinogen to produce angiotensin I (Ang I). Ang I is further cleaved by angiontensinogen converting enzyme (ACE), found primarily in the lung, leading to the formation of angiotensin II (Ang II). Ang II regulates blood pressure by both stimulating sodium transporters [56] and by increasing the production and release of aldosterone [57]. Offspring born to mothers fed a low protein diet during pregnancy go onto develop high blood pressure. We have shown that administration of ACE inhibitors in early postnatal life decreased blood pressure in the hypertensive animals [58]. When the ACE inhibitor treatment was started as early as 2 weeks postnatally, the decrease in blood pressure was maintained into adulthood, even in absence of inhibitor [59]. These results argued that the critical step for the nutritional programming was the ANGII. Further weight was added to this hypothesis when an antagonist of the ANGII receptor was administered, giving a similar response [60].

The renin–angiotensin system is one component of a complex network of systems which regulate blood pressure. The sympathetic nervous system also plays a key role, with the hypothalamus integrating signals from baro- and osmoreceptors with activity of the renin–angiotensin system, peripheral vascular resistance and renal function. There is a growing body of evidence to suggest that many aspects of this layer of control are subject to programming influences, with animals exposed to protein restriction, maternal food restriction and high fat feeding *in utero* exhibiting altered baroreceptor sensitivity [61], enhanced pressor responses to stress [62] and vasoconstrictors [63].

The effects of nutritional insults on organ differentiation and proliferation may not, of course, be directly mediated through the relative deficiency or excess of substrates needed for growth, or by direct nutrient–gene interactions. Nutrients may also impact upon embryonic and fetal development through indirect modulation of the endocrine cross-talk across the placenta. Glucocorticoids, for example, have a wide variety of functions during pregnancy, including promoting both proliferation and differentiation. In pregnancy, there is a substantial gradient of glucocorticoids across the placenta. The placenta converts glucocorticoids to their inactive form by the enzyme 11β-hydroxysteroid dehydrogenase 2 (11βHSD2) [64], a function which is critical in allowing the fetus to develop its own regulation of development. Anything that happens to reduce 11ßHSD2 activity in the placenta can result in over-exposure to glucocorticoids of maternal origin and hence inappropriate patterns of growth, gene expression and development in the fetus. Work from our laboratory demonstrated that the feeding of a low protein diet in rat pregnancy reduced placental activity of 11ßHSD by approximately one third [65] and also showed that carbenoxolone, an inhibitor of 11ßHSD2, could mimic the effect of a maternal low protein diet [66]. Programming of high blood pressure by protein restriction is to some extent dependent on the presence of maternal glucocorticoids as pharmacological ablation of steroid synthesis using metyrapone negates the effects of nutritional insult [18,67]. Others have treated rats with dexamethasone, a poor substrate for 11ßHSD2, and generated hypertension and renal defects in the offspring, which closely resemble the long-term sequelae of maternal undernutrition [68].

High concentrations of fetal glucocorticoids can also alter activity of the hypothalamic–pituitary–adrenal (HPA) axis. The HPA axis is regulated by a negative feedback system. Glucocorticoids released into the circulation by the adrenal gland interact with the glucocorticoid receptors of the pituitary, hypothalamus and hippocampus. This in turn alters responses to glucocorticoids released in future stress events. There is now evidence that exposure to excess fetal glucocorticoids at critical periods of development can alter the set point of the HPA axis leading to both altered basal and stress-induced glucocorticoid responses postnatally in both rodents and humans [69,70]. Studies of the long-term programming effects of undernutrition suggest that this can have a similar effect. Fetal exposure to maternal protein restriction results in a blunted circadian profile of ACTH secretion and is associated with programming of glucocorticoid receptor expression in a variety of tissues [71,72]. Moreover, in rats with hypertension programmed by a maternal low protein diet, adrenalectomy normalises blood pressure, whilst adrenalectomy with corticosterone replacement maintains higher pressure [73]. It is tempting to conclude that disturbances of HPA axis function may help explain fetal programming, particularly as such disturbances have long been associated with obesity and insulin resistance [74].

## The epigenetic hypothesis

Each of the proposed mechanisms described above has merits, but they do not explain what is happening on a molecular scale or fully address what may be the primary response to an adverse nutritional environment during development. It is not clear why, for example, diverse forms of undernutrition result in remodelling of the kidney, or why the same nutritional insults should bring about changes in placental 11ßHSD2 activity. Recently, evidence has been accumulating that nutritional deprivation can induce epigenetic changes, which in turn can programme the phenotype of an individual.

Epigenetics describes the study of heritable changes in gene expression that are not caused by changes in the primary DNA sequence [75]. The epigenetic code is a series of small marks added to DNA or to histone proteins. There are currently two well defined mechanisms by which this epigenetic code can be created; the addition of methyl groups to DNA cytosine bases and the post-translational modification of histone proteins by the addition of methyl or acetyl groups [76]. Methylation or histone modification can determine whether or not genes can be expressed, and hence generates additional flexibility in the genetic code, allowing one genotype to have a variety of phenotypes.

The epigenetic code, although heritable, is thought not to be fixed throughout the life stages. It is known that it is vulnerable to alteration during several life stages, spanning embryogenesis, fetal and neonatal development, puberty and old age [77,78]. There is evidence that epigenetic drift occurs across the lifespan and this can promote both DNA hypo- and hyper-methylation. The basis of age-related epigenetic drift is that there is a progressive decline in expression of DNA methyltransferase-1 (DNMT1), the enzyme which maintains DNA methylation patterns set earlier in life. This leads to passive demethylation of the whole genome. In some tissues the response to this may be up-regulation of DNA methyltransferase-3b (DNMT3b), leading to hypermethylation of CpG islands in specific gene promoters. This mechanism has been shown to explain changes in gene expression in the development of human cancers and Alzheimers disease. During gestation the developing embryo/fetus is subject to both de-methylation and re-methylation [79] and this may make an individual particularly susceptible to environmental interference, including that of nutrition as the process of methylation is dependent upon an adequate supply of folates, choline and other B vitamins.

It is now well established that diets that are suboptimal in nutrients directly involved in methyl-group metabolism can significantly alter the epigenetic code. Sinclair and colleagues demonstrated that restriction of the maternal supply of B vitamins during the periconceptual period in sheep could alter the methylation state of up to 4% of the whole genome. Even where methyl donors are present in apparently normal concentrations, undernutrition can interfere with the setting of epigenetic marks [71,80–82]. Two mechanisms are now proposed by which all nutritional, and environmental factors, can alter the epigenetic code. Alterations could be caused firstly by directly interfering with the process of DNA or histone methylation by affecting the supply of methyl donors in the diet, or the activity of the enzymes responsible for DNA methylation or histone modifications, such as acetylation and methylation.

A second possible mechanism is by changing the amount of DNA available for methylation by altering the transcriptional activity of specific genes during times of DNA methylation [76]. Lillycrop and colleagues have demonstrated that the hepatic expression of PPARα is up-regulated in young adult rats following fetal exposure to maternal protein restriction. The increased gene expression is associated with hypomethylation of the gene promoter, altered expression of DNA methyltransferases and modification of histones. Similarly the adrenal expression of the angiotensin type 1b receptor is up-regulated by maternal undernutrition, following hypomethylation of the promoter [83]. In the latter case, the effect appears dependent upon the production of maternal steroids during pregnancy, suggesting that there is some effect of antenatal glucocorticoids upon the epigenome of the developing fetus.

Although of major interest the epigenetic hypothesis leaves many unanswered questions. For example, why does a low protein diet have a specific effect on particular genes and particular tissues, rather than affecting the whole of the epigenome? Which specific component of a diet alters the epigenetic process? And, of course, what are the specific steps in the process that result in changes in epigenetic profile? It is also important to note that resetting of epigenetic marks does not explain all of the observed programming effects of nutritional stressors. Bogdarina and colleagues demonstrated that in offspring of rats fed a low protein diet during pregnancy, the hepatic expression of glucokinase was down-regulated in the absence of any changes in DNA methylation [84]. Moreover, although there are a number of reports of maternal nutrition impacting on DNA methylation and gene expression, none of the specific genes described are known to be major candidate genes for human disease processes. Despite their drawbacks, however, epigenetic explanations are very tempting as the cause of programming and it is possible that these underpin the phenotype we observe when development and nutrition intertwine. Given the potential heritability of epigenetic changes occurring within germ cells, this mechanism would also provide a useful explanation of how certain effects of undernutrition during pregnancy can be transmitted to more than one successive generation [85,86].

## The concept of the gatekeeper

Evidence to support the proposed mechanisms discussed above has been provided by a large number of experimental studies. Each of these studies has attempted to address the important aim of identifying how nutritional signals during fetal development permanently alter organ structure and function, and hence promote greater disease risk. However, all are essentially observational studies and do not explain the commonality between models, in particular the remarkable similarities between the effects of maternal undernutrition and maternal over-feeding. As such, they are unable to determine the common underlying mechanisms and principles that drive programmed disease processes. Animal studies in this field tend to characterise the downstream phenotypes that are observed in adult offspring and hence focus on processes that may mediate the exact pathology or metabolic consequences of programming. Many of these processes are likely to be secondary phenomena and do not explain the basis of programming. Broad assumptions have been drawn about observed changes in expression of single genes or specific pathways that have been selected on the basis of their plausible involvement in development of metabolic or vascular phenotypes. Close association of gene expression changes with the phenotype of interest actually increases the likelihood that observed effects are secondary events. Therefore it has to be recognised that the most likely drivers of nutritional programming are still unknown.

One of the most interesting aspects of all the work that has been carried out on experimental animals to try and understand how fetal programming may occur, is the finding of remarkable similarity between the phenotypes that arise in response to different maternal insults. For example in rats, the feeding of low protein, high fat or low iron diets in pregnancy all result in hypertension and relative obesity. This observation has given rise to what we are terming the “gatekeeper hypothesis”. The essence of this hypothesis is that there are a limited number of genes or gene pathways that are influenced by a nutritional insult and that changes to these core biological processes represent the primary response to insult from which all subsequent events leading to disease are derived.

**Fig. 1:**
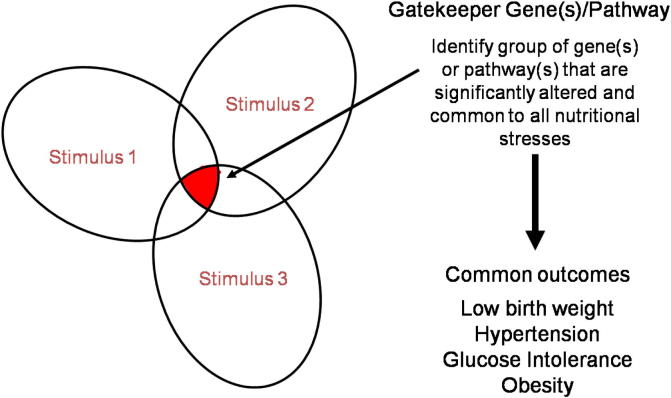
The gatekeeper hypothesis. The figure shows a Venn diagram of the genes and pathways altered by different nutritional stresses. Within each pattern is an overlap outlined in the shaded area, and we hypothesise that these common genes are the ones that are responsible for generating the phenotype.

The hypothesis can be represented graphically by the Venn diagram shown in Fig 1. In essence, any nutritional stress will generate a complex set of responses. Some of these will be homeostatic, occurring to reduce the impact of the nutritional stressor and to maintain stores and levels of critical nutrients. Potentially these responses could initiate the chain of events leading directly to postnatal pathology, but the majority may have no lasting consequence. Other responses will be pathological responses, occurring as a consequence of, or themselves causing, damage related directly to the nutritional stressor.

**Fig. 2:**
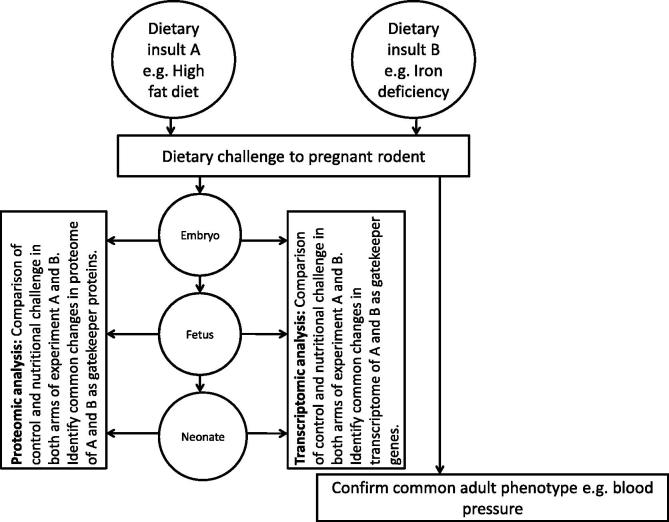
Proposed experimental design to test the gatekeeper hypothesis. The gatekeeper hypothesis asserts that relatively few common mechanisms will operate to determine the common phenotypes that follow programming insults. Testing the hypothesis therefore relies on the parallel study of two maternal dietary insults which share a common phenotypic outcome in the offspring. Use of techniques such as microarray or proteomics allows an unbiased and systematic approach to identify the molecular targets of the maternal insults. Analysis of proteomic and gene array data will identify core gatekeeper processes and pathways and provide insight into the mechanisms which drive programming across different developmental stages.

The identification of the gatekeeper processes represents a significant technical challenge, but the recent advances in proteomics and nutrigenomics puts this within the grasp of researchers in this field. The experimental approach that we propose (Fig. 2) involves the parallel application of two nutritional insults known to programme a common phenotype, such as high blood pressure, during rat pregnancy and the collection of embryonic or fetal material. Preparation of samples for proteomic and microarray analysis would generate a wealth of data from which pathway analysis would identify the genes, proteins and processes which are differentially regulated by both nutritional insults, with the change in expression/activity being in the same direction (i.e. up- or down-regulated in response to both insults). This experimental approach could be enhanced by the use of DNA methylation arrays or deep sequencing to explore whether gatekeeper processes are influenced by modifications to the epigenome.

The suggested approach for identification of gatekeeper processes has a number of associated problems. It is likely, for example, that up- or down-regulation of a fundamental process such as regulation of the cell cycle might have a profound effect even if the disturbance was extremely short-lived. Thus the point in gestation at which analysis is performed will be a critical issue. A further complication is introduced when considering where the gene changes may be located. The pattern of gene expression will vary between different tissues, so it would be expected that the changes initiated by a nutritional stress could be different in different organs. The timeframe of vulnerability may also differ between organs depending upon their relative rate of maturation and the differences in onset of organogenesis. Additionally, changes in gene expression as a consequence of nutritional stress tend to be relatively mild. This is a problem for interpretation, since array and similar technologies are not usually very sensitive. The difficulties can be minimised by robust statistical analysis and careful experiment design.

Importantly, it may not be identical genes that are affected by the stress in each modality. Instead, it may be that different genes along the same pathway, or impacting on the same basic process, may be altered. Thus, examination of the data sets using pathway analysis software is essential. Currently, we are testing the hypothesis using two nutritional stressors (protein and iron restriction) but hope to add further gene sets as they become available.

## Conclusion

The observation that there is commonality in the long-term programming response to a diverse range of nutritional insults in pregnancy provides an important opportunity to determine the common fundamental biological processes which underpin the developmental origins of adult disease. The challenge, of course, is to design appropriate experimental approaches to elucidate the relevant processes!

## Conflicts of interest

The authors have no conflicts of interest to declare.
